# Influence of Head Motion on the Accuracy of 3D Reconstruction with Cone-Beam CT: Landmark Identification Errors in Maxillofacial Surface Model

**DOI:** 10.1371/journal.pone.0153210

**Published:** 2016-04-11

**Authors:** Kyung-Min Lee, Jin-Myoung Song, Jin-Hyoung Cho, Hyeon-Shik Hwang

**Affiliations:** 1 Department of Orthodontics, School of Dentistry, 4D Dental Research Institute, Chonnam National University, Gwangju, Korea; 2 Department of Orthodontics, School of Dentistry, Dental Science Research Institute, Chonnam National University, Gwangju, Korea; University of North Carolina at Chapel Hill, UNITED STATES

## Abstract

**Purpose:**

The purpose of this study was to investigate the influence of head motion on the accuracy of three-dimensional (3D) reconstruction with cone-beam computed tomography (CBCT) scan.

**Materials and Methods:**

Fifteen dry skulls were incorporated into a motion controller which simulated four types of head motion during CBCT scan: 2 horizontal rotations (to the right/to the left) and 2 vertical rotations (upward/downward). Each movement was triggered to occur at the start of the scan for 1 second by remote control. Four maxillofacial surface models with head motion and one control surface model without motion were obtained for each skull. Nine landmarks were identified on the five maxillofacial surface models for each skull, and landmark identification errors were compared between the control model and each of the models with head motion.

**Results:**

Rendered surface models with head motion were similar to the control model in appearance; however, the landmark identification errors showed larger values in models with head motion than in the control. In particular, the Porion in the horizontal rotation models presented statistically significant differences (*P* < .05). Statistically significant difference in the errors between the right and left side landmark was present in the left side rotation which was opposite direction to the scanner rotation (*P* < .05).

**Conclusions:**

Patient movement during CBCT scan might cause landmark identification errors on the 3D surface model in relation to the direction of the scanner rotation. Clinicians should take this into consideration to prevent patient movement during CBCT scan, particularly horizontal movement.

## Introduction

Cone-beam computed tomography (CBCT) has been widely used in dentistry since its introduction to dentistry in 1998 [1]. The applications of CBCT images include for the management of impacted tooth [2], dental measurements [3], evaluation of root resorption [4], diagnosis of temporomandibular joint [5,6], airway assessment [7], orthognathic surgery [8], and surgical evaluation [9].

CBCT scan data are obtained during a single rotation of the source-detector around the patient’s head. Projection images taken from different angles are reconstructed to form three-dimensional (3D) volume data through modification of the original cone-beam algorithm developed by Feldkamp [10]. When a patient moves during the CBCT scan, misregistration of data voxels occurs, which can influence the reconstruction process of all projection data and presents as shading or streaking in the reconstructed image [11]. The acquisition time of CBCT machines ranges roughly between 6 and 20 seconds, which is enough time for a patient’s head to experience minor movement [12]. Donaldson et al [13] assessed 200 CBCT scans taken between 2008 and 2010 in Glasgow Dental Hospital and School. The authors found increased motion artifacts in the patients under 16 years and above 65 years of age.

Patient movement may result in motion artifacts such as blurring, doubling, and defects, which can appear in medical imaging not exclusive to CT [14,15], including other imaging modalities such as magnetic resonance imaging (MRI) [16], positron emission tomography (PET) [17], and single-photon emission tomography (SPECT) [18]. Marco et al [19] investigated the effects of head movement on reconstructed image quality in relation to patient positioning in CBCT systems. Their results indicated that patient movement can significantly affect resolution of the final image and that acquisition while lying down may be preferred to reduce the detrimental effects of motion on CBCT image quality. Since artifacts such as blurring or doubling degrade the quality of images, motion artifacts make it difficult to perform identification of anatomic structures and anatomical landmarks-based registration for image-guided surgery [20]. The purpose of the present study was to investigate the influence of head motion on the accuracy of landmark identification on the maxillofacial 3D surface model.

## Materials and Methods

**Fig 1:**
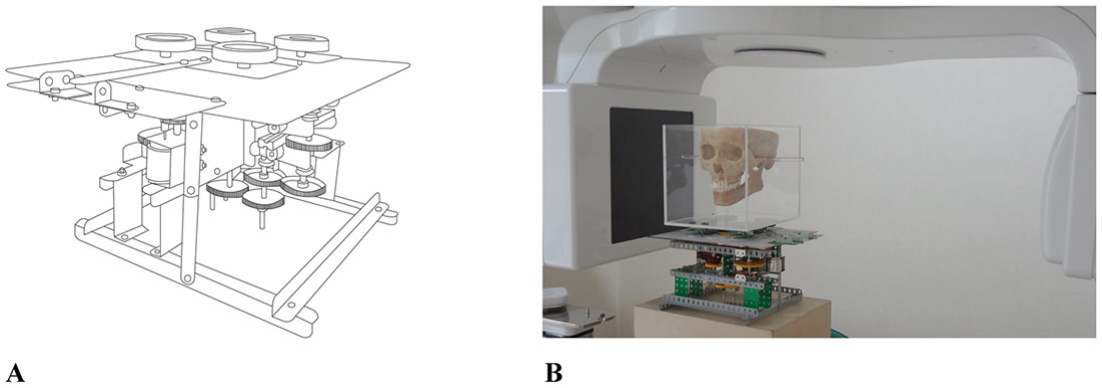
The motion controller fabricated in this study. A dry human skull was mounted on the motion controller. Four types of head motion were simulated by remote control.

Fifteen dry human skulls from the Department of Oral Anatomy at the School of Dentistry of Chonnam National University were included in this study. The present study was exempted from approval by the Chonnam National University Dental Hospital Institutional Review Board (CNUDH-EXP-2015-001). A motion controller was fabricated to simulate head motions of the skull during CBCT scan. Acrylic box was mounted on the motion controller for placement of skull. Four types of head movements were simulated by remote control outside the CBCT room. The skull was placed in the acrylic box and was incorporated into the motion controller. Then acrylic ear rods were positioned on the left and right external auditory canals of the dry skull to construct an axis for the vertical rotation. CBCT scans were obtained with Alphard Vega (Asahi Roentgen Co., Kyoto, Japan) under the following conditions: 80kV, 5mA, voxel size of 0.39 × 0.39 × 0.39 mm, and field of view (FOV) of 200 × 179 mm. An x-ray emitter rotated a full 360 degrees around the subject with 17 seconds of the scanning time. In addition, the CBCT data were transmitted within 11 seconds, and the slice images were created and produced within 90 seconds. During the scan, four different types of head motion were triggered to occur at the start of the scan using remote control: 2 horizontal rotations (to the right/to the left) and 2 vertical rotations (upward/downward) (Fig 1). The head was rotated 10° for one second and was not returned to its original position. Five different images were obtained from each skull: no motion as a reference, and head motion with 10° right rotation, 10° left rotation, 10° upward rotation, and 10° downward rotation. Based on the pilot test and the previous study [21], 10° was determined as the minimal movement range of the skull during CBCT scan.

### Three-dimensional surface model reconstruction and landmark identification

CBCT scan data for each skull were exported to Invivo5^™^ software (version 5.2, Anatomage, San Jose, CA, USA) as a digital imaging and communication in medicine (DICOM) file. Using the *volume rendering* function, a 3D surface model was created automatically and visualized in the *Bone* mode. The default setting values were optimized for each skull.

To reorient the skull in the standard position, the point of nasion (the most anterior point on the frontonasal suture) was set at zero (x, y, and z = 0, 0, and 0) in the 3D coordinate system using the patient orientation function of the software program. According to the software, the x-, y- and z-axes indicate the mediolateral, anteroposterior, and superoinferior axes, respectively.

To evaluate the effect of head motion during CBCT scan on the accuracy of the landmark identification in the 3D surface model, nine landmarks (three midline landmarks: the Crista galli, Anterior nasal spine, and Menton, and three pairs of bilateral landmarks: the Porion, Gonion lateralis, and Gonion inferius), which are commonly used in orthodontics and maxillofacial surgery, were selected for the study (Table 1). The primary examiner (K.M.L.) repeated the landmark identification after two weeks and then the 3D landmark coordinates for each movement were obtained. The examiner was previously trained to use the Invivo5^™^ software and to identify landmarks on surface models in over 100 cases. The x-, y-, and z-coordinates of the landmarks were recorded, and the difference between the first and second measurement trials was calculated in three-dimensions. The Euclidean distance, which is the square root of the sum of the squared coordinate differences between the two identified positions, was calculated for each pair of repeated measures. The secondary examiner (J.M.S) identified all landmarks and inter-examiner reproducibility was tested with the intra-class correlation coefficient (ICC) using a 2-way random-effects model with absolute agreement.

**Table 1:** Definition of three-dimensional landmarks used in this study.

*Landmark*	*Abbreviation*	*Definition*
Crista galli	Cg	Most superior point of the cribriform plate of the ethmoid bone
Anterior nasal spine	ANS	Most anterior point of the anterior nasal spine
Menton	Me	Most inferior point on the symphysis
Porion	Po	Most superior point of the external acoustic meatus
Gonion lateralis	Go_lat_	Most lateral point on the gonion area
Gonion inferius	Go_inf_	Most inferior point on the gonion area

To compare the landmark identification errors between the control model and each of the models with head motion, a paired *t*-test was used for each landmark. In addition, the mean and standard deviation was computed for each coordinate direction in order to evaluate which direction of error contributed to the degree of overall error. Paired *t*-tests were used to determine the differences in the errors in three-dimensions between the control model and each of the head motion models. In the case of bilateral landmarks, the identification errors between the right and left sides were compared with the paired *t*-test. In addition, the intraobserver reliability was assessed by calculating the intraclass correlation coefficients (ICCs). Statistical evaluations were performed at the 5% level of significance with SPSS software (version 17.0, SPSS, Chicago, IL, USA).

The sample size of this study was not calculated a priori, but, the post hoc power analysis by the G*power program (version 3.1.9.2, Department of Experimental Psychology, Heinrich-Heine-University, Dusseldorf, Germany) [22] showed over 90% power for all the measurements.

## Results

**Fig 2:**
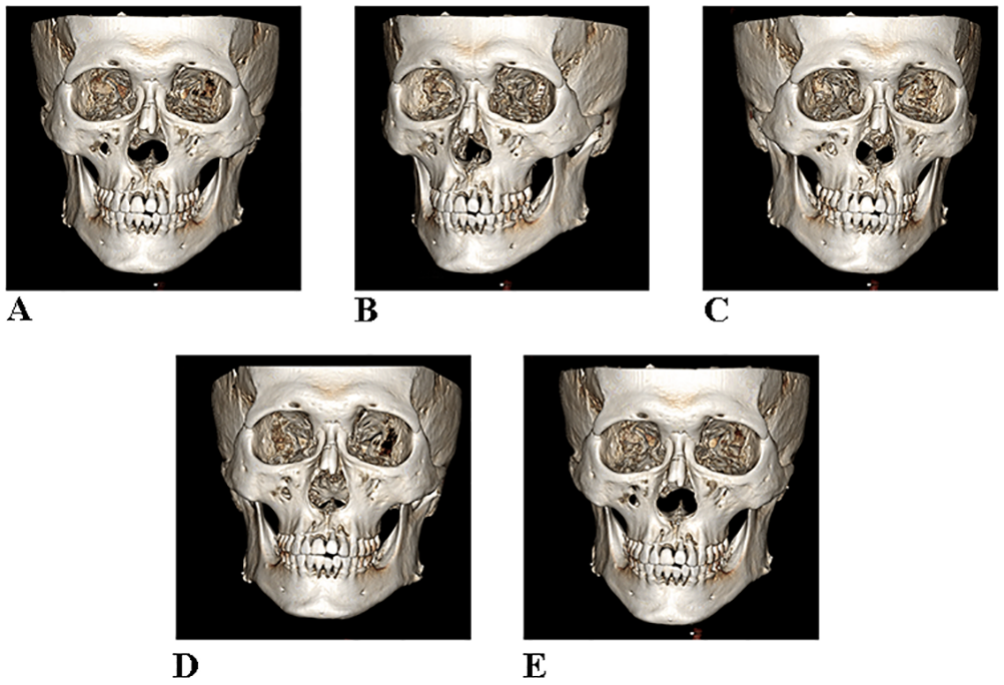
Rendered 3D surface models without motion (A) and with head motion (B-E). Although head motion occurred during the scan, the surface models look like to the control model in appearance. **A**, No motion; **B**, right side rotation; **C**, left side rotation; **D**, upward rotation; **E**, downward rotation.

A visual examination of rendered 3D surface models revealed that the head motion models were similar to the control model in appearance. Each head orientation was different from the control image, but the image quality looked same as in the control (Fig 2).

The intraexaminer reliability values for the x, y, and z coordinates of most landmarks in the control and head motion groups were greater than 0.9. Only the right and left Porions had intraexaminer reliability values for the x-axis (0.837 and 0.842, respectively) in the models with right head rotation, and for the x-axis (0.811 and 0.792, respectively) in the models with left head rotation.

The landmark identification errors were different from those of the control model. Table 2 shows the mean differences between the initial and repeated identifications of each landmark by the same examiner as the Euclidian distance. The errors showed larger values in the head motion models than in the control. Bilateral landmarks (Porion, Gonion lateralis, and Gonion inferius) showed relatively large errors compared to the midline landmarks (Crista galli, Anterior nasal spine, and Menton). In particular, the Porion in the right and left side rotation models presented a statistically significant difference from those in the control model. On the other hand, in the case of upward and downward rotation models, all landmark identification errors presented no statistically significant differences compared with the control model (Table 2).

**Table 2:** Landmark identification errors (mm) in Euclidean distance of the primary examiner for each head motion and comparison with the control model (N = 15).

	Control	Right	Left	Upward	Downward
	Mean ± SD	Mean ± SD	Mean ± SD	Mean ± SD	Mean ± SD
**Midline**					
**Cg**	0.5 ± 0.4	0.5 ± 0.4	0.4 ± 0.3	0.6 ± 0.3	0.6 ± 0.2
**ANS**	0.5 ± 0.3	0.4 ± 0.2	0.6 ± 0.3	0.6 ± 0.3	0.5 ± 0.2
**Me**	0.6 ± 0.2	0.9 ± 0.2	0.8 ± 0.5	0.6 ± 0.4	0.8 ± 0.3
**Right side**					
**Po**	0.7 ± 0.3	1.0 ± 0.4*	1.1 ± 0.1*	0.7 ± 0.3	0.9 ± 0.2
**Go**_**lat**_	0.5 ± 0.3	0.6 ± 0.4	0.9 ± 0.2	0.9 ± 0.4	0.6 ± 0.3
**Go**_**inf**_	0.5 ± 0.3	0.8 ± 0.3	0.9 ± 0.3	0.8 ± 0.3	0.7 ± 0.5
**Left side**					
**Po**	0.8 ± 0.1	1.4 ± 0.5*	1.4 ± 0.1*	0.7 ± 0.4	0.8 ± 0.4
**Go**_**lat**_	0.7 ± 0.4	0.8 ± 0.3	1.0 ± 0.2	0.7 ± 0.2	0.6 ± 0.3
**Go**_**inf**_	0.6 ± 0.2	0.9 ± 0.5	1.1 ± 0.4	0.9 ± 0.5	0.7 ± 0.4

*Right*, right side rotation; *Left*, left side rotation; *Upward*, upward rotation; *Downward*, downward rotation; *SD*, standard deviation.

*P < 0.05 by the paired *t*-test. Unit is mm.

In order to evaluate which direction of error contributed to the overall error, the mean and standard deviation was computed for each coordinate direction. Table 3 shows the mean differences between the initial and repeated identifications of each landmark by the same examiner in the x-, y-, and z-axes. In the right and left side rotation models, for the Porion, which showed the largest value in overall error, the x-direction error showed a statistically significant difference compared to the control model, indicating that the large identification errors in the Porion were attributed mostly to the mediolateral direction error (Table 3).

**Table 3:** The primary examiner’s landmark identification errors (mm) in 3-dimension of each head motion and comparison with the control model (N = 15).

	Control	Right	Left	Upward	Downward
	Mean ± SD	Mean ± SD	Mean ± SD	Mean ± SD	Mean ± SD
**x-direction**					
**Midline**					
**Cg**	0.1 ± 0.1	0.3 ± 0.9	0.2 ± 0.1	0.3 ± 0.2	0.2 ± 0.1
**ANS**	0.2 ± 0.2	0.1 ± 0.2	0.5 ± 0.4	0.2 ± 0.2	0.1 ± 0.4
**Me**	0.5 ± 0.3	0.3 ± 0.3	0.5 ± 0.3	0.4 ± 0.3	0.2 ± 0.4
**Right side**					
**Po**	0.2 ± 0.2	0.9 ± 0.3*	0.8 ± 0.1*	0.4 ± 0.3	0.4 ± 0.4
**Go**_**lat**_	0.2 ± 0.2	0.2 ± 0.2	0.1 ± 0.2	0.2 ± 0.3	0.2 ± 0.2
**Go**_**inf**_	0.1 ± 0.2	0.3 ± 0.3	0.5 ± 0.4	0.2 ± 0.1	0.3 ± 0.4
**Left side**					
**Po**	0.3 ± 0.3	0.8 ± 0.2*	0.8 ± 0.1*	0.2 ± 0.3	0.2 ± 0.1
**Go**_**lat**_	0.4 ± 0.4	0.2 ± 0.1	0.4 ± 0.2	0.2 ± 0.3	0.2 ± 0.1
**Go**_**inf**_	0.2 ± 0.3	0.6 ± 1.3	0.2 ± 0.3	0.4 ± 0.7	0.1 ± 0.3
**y-direction**					
**Midline**					
**Cg**	0.2 ± 0.1	0.2 ± 0.3	0.4 ± 0.2	0.4 ± 0.3	0.8 ± 0.2
**ANS**	0.2 ± 0.3	0.4 ± 0.8	0.6 ± 0.7	0.1 ± 0.3	0.4 ± 0.8
**Me**	0.2 ± 0.1	0.2 ± 0.3	0.1 ± 0.2	0.2 ± 0.2	0.1 ± 0.1
**Right side**					
**Po**	0.4 ± 0.3	0.4 ± 0.3	0.4 ± 0.2	0.4 ± 0.2	0.3 ± 0.4
**Go**_**lat**_	0.6 ± 0.9	0.4 ± 0.3	0.4 ± 0.4	0.3 ± 0.3	0.1 ± 0.1
**Go**_**inf**_	0.6 ± 0.4	0.4 ± 0.5	0.4 ± 0.4	0.4 ± 0.3	0.5 ± 0.3
**Left side**					
**Po**	0.4 ± 0.6	0.3 ± 0.1	0.6 ± 0.2	0.5 ± 0.3	0.1 ± 0.2
**Go**_**lat**_	0.8 ± 0.7	0.7 ± 0.4	1.0 ± 1.0	0.7 ± 0.9	0.7 ± 0.8
**Go**_**inf**_	0.4 ± 0.3	0.5 ± 0.6	0.5 ± 0.5	0.6 ± 1.0	0.6 ± 0.4
**z-direction**					
**Midline**					
**Cg**	0.4 ± 0.4	0.1 ± 0.3	0.1 ± 0.3	0.4 ± 0.4	0.4 ± 0.6
**ANS**	0.3 ± 0.3	0.4 ± 0.3	0.9 ± 1.3	0.5 ± 0.2	0.3 ± 0.3
**Me**	0.1 ± 0.3	0.6 ± 0.9	0.3 ± 0.3	0.6 ± 0.4	0.4 ± 0.6
**Right side**					
**Po**	0.2 ± 0.2	0.4 ± 0.3	0.4 ± 0.5	0.1 ± 0.1	0.2 ± 0.2
**Go**_**lat**_	0.3 ± 0.2	0.4 ± 0.3	0.3 ± 0.2	0.3 ± 0.3	0.6 ± 0.1
**Go**_**inf**_	0.1 ± 0.3	0.1 ± 0.3	0.1 ± 0.3	0.3 ± 0.4	0.1 ± 0.3
**Left side**					
**Po**	0.4 ± 0.5	0.7 ± 1.2	0.7 ± 0.7	0.4 ± 0.3	0.2 ± 0.1
**Go**_**lat**_	0.5 ± 0.4	0.3 ± 0.3	0.2 ± 0.2	0.5 ± 0.8	0.5 ± 0.5
**Go**_**inf**_	0.3 ± 0.4	0.8 ± 1.5	0.4 ± 0.4	0.3 ± 0.6	0.3 ± 0.4

*Right*, right side rotation; *Left*, left side rotation; *Upward*, upward rotation; *Downward*, downward rotation; *SD*, standard deviation.

*P < 0.05 by the paired *t*-test.

**Fig 3:**
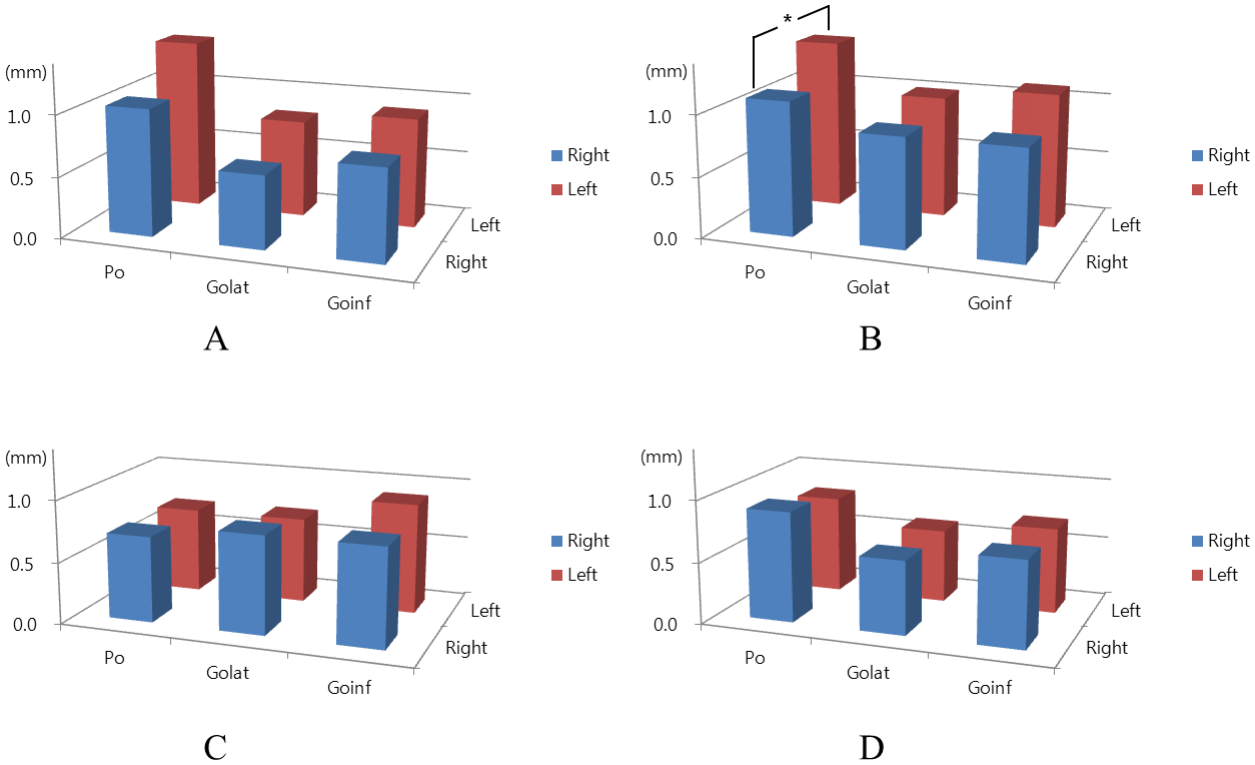
The primary examiner’s landmark identification errors in bilateral landmarks and comparison between the right and left sides. **A**, Right side rotation; **B**, left side rotation; **C**, upward rotation; **D**, downward rotation. In the horizontal rotation models, the errors of left side landmarks showed higher values than the right side. In particular, Porion showed statistical significances. On the other hand, there were no significant differences between the right and left side landmarks in the vertical rotation models. *P < 0.05 by the paired *t*-test.

In order to evaluate the landmark identification errors in the bilateral landmarks, the errors were compared between right and left sides. In the horizontal rotation models, the errors were higher in the left side rotation than in the right side rotation. In the left side rotation model, the errors of left side landmarks showed higher values than the right side overall. In particular, Porion showed a statistically significant difference between the right and left sides. On the other hand, there were no significant differences between the right and left landmarks in the vertical rotation models (Fig 3). Regarding reproducibility, the interexaminer landmark identification errors in Euclidean distance were highly correlated (Table 4).

**Table 4:** Interexaminer reproducibility of landmark identification errors in Euclidean distance.

	Control	Right	Left	Upward	Downward
	ICC	ICC	ICC	ICC	ICC
**Midline**					
**Cg**	0.938	0.925	0.939	0.962	0.907
**ANS**	0.925	0.929	0.901	0.923	0.954
**Me**	0.928	0.878	0.803	0.864	0.939
**Right side**					
**Po**	0.803	0.831	0.827	0.801	0.835
**Go**_**lat**_	0.824	0.804	0.807	0.793	0.822
**Go**_**inf**_	0.837	0.799	0.797	0.824	0.837
**Left side**					
**Po**	0.818	0.779	0.793	0.804	0.874
**Go**_**lat**_	0.833	0.829	0.871	0.864	0.889
**Go**_**inf**_	0.842	0.801	0.796	0.873	0.869

*Right*, right side rotation; *Left*, left side rotation; *Upward*, upward rotation; *Downward*, downward rotation.

## Discussion

An accurate 3D surface model of the maxillofacial structure is essential to make a diagnosis and to establish a treatment plan such as surgical simulation in the computer-assisted surgery. Sun et al [23] reported that anatomical landmarks in the anterior maxilla and zygomatic region might be used for orthognathic surgery to assess occlusal changes during surgery. The landmarks selected in this study were those commonly used for 3D volume superimposition. Volume superimposition is useful for assessing the treatment changes between before and after treatment CBCT scans. For volume superimposition of CBCT scans, the original volume is registered to the second volume by selecting stable landmarks. It is critical to identify the anatomical landmarks correctly for accurate volume registration. Motion artifacts can cause image blurring or unclear margins of anatomic structures, resulting in an increase of the landmarks identification errors.

The motion controller used in the present study was fabricated to allow four different types of quantitative head rotation. It could rotate the skull upward or downward with an imaginary line connecting the right and left ear rods as the axis of rotation, as well as rotate it sideways to the right or left with the vertical axis which passed through the center of the imaginary line connecting the right and left ear rods as the axis of rotation. Particularly, it was designed to simulate various types of rotations with specific angles for a certain time, enabling quantification of head motion.

The Euclidean distance between the two repeated landmark coordinates was calculated between the two points in 3D space. Although there is no standard for acceptable error in landmark identification, the available literature on 2D cephalometrics and 3D reconstructions for geometric and morphometric analysis has reported acceptable measurement errors to be less than 1.0 mm [24]. In the present study, the greatest Euclidean distances were observed for the left Porion (1.4 mm) of the models with right and left side rotation. The next largest Euclidean distance was observed for the right Porion (1.1 mm) of the left side rotation model, followed by right Porion (1.0 mm) of the right side rotation model. In both the right and left side rotation models, the Porion showed a statistically large value of error in comparison with the control model. The reason why Porion showed largest errors among the landmarks is likely that its surrounding bone has low density. In addition, the Porion is located far from the axis of horizontal head rotation whereas it is close to the axis of vertical rotation. The results of Porion identification errors in 3-dimension also revealed that most errors in the right and left side rotation models occurred in x-direction.

Compared with right structures, the left structures showed a larger value of identification errors on the models with horizontal rotation. Considering the direction of the CBCT scan and subject movement, the left side of the maxillofacial structure may be more blurred than the right side when subject movement occurs at the beginning of the CBCT scan. The CBCT scanner starts scanning in front of the subject and rotates a full 360 degrees, in other words, from the subject’s right side to the left. In addition, left side head rotation which was opposite direction to the scanner rotation may influence the inconsistencies of projection data more than right side head rotation. In clinical practice, clinicians should take this into consideration to prevent patient movement at the start of a CBCT scan, and clinicians must pay attention to the patient’s horizontal movement, particularly left side head rotation.

In the present study, head motion was given to 10 degrees for one second at the beginning of the CBCT scan. Hanzelka et al [25] found that patient movement was significantly higher at the beginning of the scan, when noise and vibrations were likely to surprise the patient. Various conditions during CBCT scanning and subject movement, according to amount and duration of movement, as well as the timing of the onset of movement, can affect the occurrence of motion artifacts and image quality. Further research related to the various conditions of movement is currently in progress.

Since patient movement during CBCT scanning may significantly influence image quality, it would be effective to restrict patient movement during the CBCT scan. However, the assumption of complete motionlessness is impractical in clinical application because the motion of some human organs is unavoidable, such as peristalsis, heart beating and other physiological motilities. Any way to reduce the patient movement should be considered. For facial soft tissue evaluation in orthodontics and maxillofacial orthognathic surgery, upper lip and chin rests should not be considered. We suggest using the forehead rest as the only head-positioning device during CBCT scanning. In addition, the patient should be asked to close his or her eyes. An occipital head rest away from the field of interest will stabilize the head without obliterating the soft tissues of the lower third of the face.

With regard to the advance of software, corrections compensating for subject movement in projection data would be required to improve the quality of images and to reduce the CBCT re-scan. Some image artifacts have already been successfully suppressed through the use of more sophisticated projection and back projection techniques [26,27]. All these methods, however, require massive computational power, a limitation which has prevented them from being used in commercial CBCT scanners for daily routine work. The results of the present study can be a further help in establishing more modern approaches to avoid reconstruction errors of CBCT scan data and can be a basis for the development of motion correction or compensation algorithms for clinical application.

## Conclusions

Landmark identification errors in the 3D surface model are affected by patient movement during CBCT scan, particularly on areas with low bone density. The errors showed an increasing tendency in the opposite direction of the scanner rotation. Clinicians should take this into consideration to prevent patient movement at the start of the CBCT scan, and they must pay attention to the patient’s horizontal movement, particularly left side head rotation.
